# Systemic elucidation on the potential bioactive compounds and hypoglycemic mechanism of *Polygonum multiflorum* based on network pharmacology

**DOI:** 10.1186/s13020-020-00401-2

**Published:** 2020-11-18

**Authors:** Yunfei Song, Jianbo Yang, Wenguang Jing, Qi Wang, Yue Liu, Xianlong Cheng, Fei Ye, Jinying Tian, Feng Wei, Shuangcheng Ma

**Affiliations:** 1 School of Chinese Materia Medica, Beijing University of Chinese Medicine, Beijing, 102488 China; 2 Institute for Control of Chinese Traditional Medicine and Ethnic Medicine, National Institutes for Food and Drug Control, Beijing, 100050 China; 3 Institute of Materia Medica, Chinese Academy of Medical Sciences and Peking Union Medical College, Beijing, 100050 China

**Keywords:** *Polygonum multiflorum*, Diabetes, Systematic pharmacology, Bioactive compounds, Hypoglycemic mechanism

## Abstract

**Background:**

Diabetes is a complex metabolic disease characterized by hyperglycemia, plaguing the whole world. However, the action mode of multi-component and multi-target for traditional Chinese medicine (TCM) could be a promising treatment of diabetes mellitus. According to the previous research, the TCM of *Polygonum multiflorum* (PM) showed noteworthy hypoglycemic effect. Up to now, its hypoglycemic active ingredients and mechanism of action are not yet clear. In this study, network pharmacology was employed to elucidate the potential bioactive compounds and hypoglycemic mechanism of PM.

**Methods:**

First, the compounds with good pharmacokinetic properties were screened from the self-established library of PM, and the targets of these compounds were predicted and collected through database. Relevant targets of diabetes were summarized by searching database. The intersection targets of compound-targets and disease-targets were obtained soon. Secondly, the interaction net between the compounds and the filtered targets was established. These key targets were enriched and analyzed by protein–protein interactions (PPI) analysis, molecular docking verification. Thirdly, the key genes were used to find the biologic pathway and explain the therapeutic mechanism by genome ontology (GO) and kyoto encyclopedia of genes and genomes (KEGG) analysis. Lastly, the part of potential bioactive compounds were under enzyme activity inhibition tests.

**Results:**

In this study, 29 hypoglycemic components and 63 hypoglycemic targets of PM were filtrated based on online network database. Then the component-target interaction network was constructed and five key components resveratrol, apigenin, kaempferol, quercetin and luteolin were further obtained. Sequential studies turned out, AKT1, EGFR, ESR1, PTGS2, MMP9, MAPK14, and KDR were the common key targets. Docking studies indicated that the bioactive compounds could stably bind the pockets of target proteins. There were 38 metabolic pathways, including regulation of lipolysis in adipocytes, prolactin signaling pathway, TNF signaling pathway, VEGF signaling pathway, FoxO signaling pathway, estrogen signaling pathway, linoleic acid metabolism, Rap1 signaling pathway, arachidonic acid metabolism, and osteoclast differentiation closely connected with the hypoglycemic mechanism of PM. And the enzyme activity inhibition tests showed the bioactive ingredients have great hypoglycemic activity.

**Conclusion:**

In summary, the study used systems pharmacology to elucidate the main hypoglycemic components and mechanism of PM. The work provided a scientific basis for the further hypoglycemic effect research of PM and its monomer components, but also provided a reference for the secondary development of PM.

## Background

Diabetes, a common chronic metabolic disease of endocrine system, is characterized by hyperglycemia. It is generally believed that diabetes is caused by a lack of insulin secretion activity or insulin resistance. Usually diabetes can be simply divided into type 1 diabetes mellitus (T1DM) and type 2 diabetes mellitus (T2DM) due to different pathogenesis. As a result of the long-term existence of hyperglycemia, it often accompanied by a variety of diabetic complications [1], such as diabetes cardiomyopathy [2], diabetic ketoacidosis [3], diabetic gastroparesis [4], retinopathy [5], diabetic nephropathies [6]. These diabetes-related diseases not only have a huge impact on the body and spirit of the patients, but also bring great pressure to society. Recently, the international diabetes federation (IDF) announced that the number of people with diabetes is on the rise with an average global growth rate of 51% around the world. Currently, there are 463 million people with diabetes, and there will be 700 million diabetic patients in the whole world by the year of 2045 (https://www.diabetesatlas.org/en/resources/). Diabetes mellitus has become the third most serious chronic non-communicable disease after cancer, cardiovascular and cerebrovascular diseases, and has become a serious public health problem which has been put into much attention [7].

Up to now, a great deal of effort and much money have been devoted to developing drugs to treat diabetes, and many drugs have been successfully developed for different targets. Some drugs targeting glucosidase [8], glucagon-like peptide-1 (glp-1) receptor [9], dipeptidase IV (dpp-4) [10], and sodium glucose co-transporter 2(SGLT2) [11] and the like have been already available on market [12]. And some other related to G protein-coupled receptor 119 (GPR119), protein tyrosine phosphatase-1b (PTP1B), glucagon receptor (GCGr), G protein-coupled receptor 40 (GPR40), glycogen synthase kinase 3 (gsk-3), glycogen phosphorylase (GP), and 11-hydroxysteroid dehydrogenase 1 (11-hsd1) are being developed. However, drugs designed for a single target sometimes couldn’t contribute to great therapeutic effect because of the complex physical reactions that diabetes brings. Traditional Chinese medicine (TCM) is considered as a complex system due to its intricate components, which correspond to complex organic targets. The system network based on the interaction between multiple components and multiple targets will help to illuminate the complicated mechanism of TCM in the treatment of diseases.

Polygonum multiflorum Thunb. (PM), a classical herbal medicine, has been used for several centuries for its functions of hair-promoted, liver-tonifying and anti-aging [13, 14]. Previous reports have shown that extracts of PM and its compounds had hypoglycemic effects [15–17]. However, researches on the hypoglycemic properties of PM are still limited and scarce. Therefore, the objective of this study is to elucidate the bioactive ingredients of PM correlated to the hypoglycemic effect and the mechanism of PM on the targets of diabetes. It is of great significance to the secondary development of PM and the further discovery of small molecule antidiabetes ligands.

Network pharmacology [18], an emerged discipline based on systems biology, has been recognized as a promising method to achieve an integral view of complex systems interacting with multiple targets of diseases. This approach coincides with the theory that TCM emphasizes the diagnosis and treatment of diseases from a comprehensive perspective and the synergy among its active compounds of TCM. Consequently, systematic pharmacology has been used to identify the key active constituents, screen the main effective integrates of TCM, investigate the disease-related key targets and biological functions and predict the potential synergetic mechanisms against complicated diseases [19, 20].

**Fig. 1:**
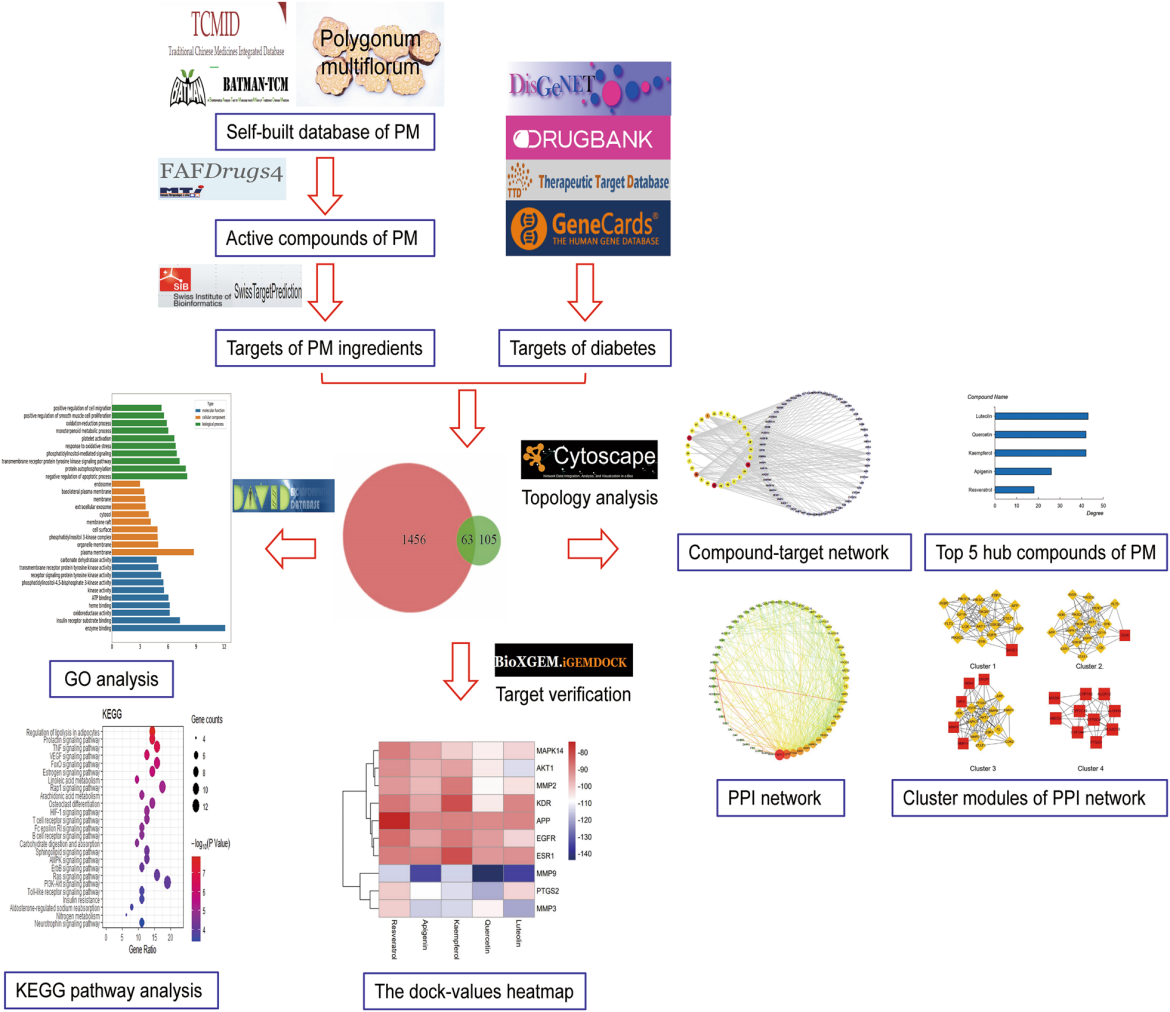
The workflow of current network pharmacology study

In the paper, systematic pharmacology was employed to discover the main constituents of PM against diabetes, to screen the core targets terms and to illuminate the crucial corresponding pathway mechanism. First, the compounds with good pharmacokinetic properties were screened from the self-established library of PM, and the targets of these compounds were predicted and collected through the database. Relevant targets of diabetes and related diseases were summarized by searching databases. The intersection targets of compound-targets and disease-targets were obtained soon. Secondly, the interaction net between the compounds and the filtered targets was established. These key targets were enriched and analyzed by PPI analysis, molecular docking verification and GO analysis. Finally, the key genes were used to find the biologic pathway and explain the therapeutic mechanism by KEGG analysis. The workflow was summarized in Fig. 1.

## Materials and methods

### Construction of PM compounds database

The ingredients of PM were gathered together from TCMID (https://www.megabionet.org/tcmid/), BATMAN-TCM platform and literature analysis related to PM [21]. Then all the chemical molecular structures and the Smiles numbers were verified and obtained by the platforms such as Pubchem (https://pubchem.ncbi.nlm.nih.gov/), Chembank (https://chembank.broadinstitute.org/), or Scifinder (https://scifinder.cas.org/) [22, 23].

### Screening of active ingredients of PM

In order to select the compounds which have better pharmacokinetic properties and oral bioavailability in vivo, the compounds were filtrated by the principle of “drug-like soft” in FAFDrugs4 (https://fafdrugs4.rpbs.univ-paris-diderot.fr/). the active ingredients were picked out when the evaluation results were “accepted”. The rule of “drug-like soft” contains the restriction to molecular weight, logP, Hydrogen Bond Acceptors (HBA), Hydrogen Bond Donnors (HBD), topological Polar Surface Area (tPSA), Number of Rotatable Bonds, rigidBonds, het/carbon atoms ratio (H/C ratio) of compounds properties and the like. The parameters range of rules were specifically in Table 1.

**Table 1:** The parameters of “Drug-Like Soft” rule

Property	Range	Property	Range
MW	100–600	Rings	≤ 6
logP	− 3 to 6	Max size system ring	≤ 18
HBA	≤ 12	Carbons	3–35
HBD	≤ 7	Hetero atoms	1–15
tPSA	≤ 180	H/C ratio	0.1–1
Rotatable bonds	≤ 11	Charges	≤ 4
Rigid bonds	≤ 30	Total charge	− 4 to 4

### Prediction of compounds targets

The targets of filtrated compounds were predicted by using the SWISS TARGET PREDICTION (STP) [24] database and SIMILARITY ENSEMBLE APPROACH (SEA) library [25]. Setting the organism “ homo sapiens”, targets with a probability value greater than 0.25 were considered as potential effective targets for these components in STP database. SEA library was used as a reference.

### Diabetes-related targets collection

The diabetes-related targets were searched in the Therapeutic Target Database (TTD) [26], Drugbank (https://www.drugbank.ca/), Disgenet (https://www.disgenet.org/) and Genecards platform (https://www.genecards.org/) with “diabetes”, “diabetes mellitus” etl. as the keywords [27]. Then these collected targets were merged and removed duplication.

### Compound-target network construction

In order to clarify the hypoglycemic targets of PM, the intersection of compound action targets and diabetes disease targets were taken as the key hypoglycemic targets of PM. Cytoscape 3.2.1 was used to establish the network diagram between the effective compounds of PM and the key diabetes targets. And the network topological properties were analyzed and the results were visualized. In the topological analysis, the degree of nodes and the betweenness centrality were significant indexes to judge the importance of nodes. The larger the degree of nodes, the more biological functions they participated in, and the more vital it was in the network. The betweenness centrality of nodes in the network reflected the closeness of the relationship between nodes. The larger the betweenness centrality, the more important the node was in the network. According to topological analysis, the key active components and main disease targets were screened.

### Protein–protein interaction analysis

The intersection targets were input into the String database to obtain the interaction results among the targets [28], and the visual analysis results were obtained by Cytoscape 3.2.1. Cytoscape-hubba was used to screen hub targets and Cytoscape-clusterONE was used for cluster analysis.

### Molecular docking verification

To verify the effectiveness of the compounds screened, molecular docking simulation was carried out between the key active compounds and the target proteins. The corresponding proteins of top 10 hub target genes were obtained in Protein Data Bank (PDB). Then the PDB format of protein was stored after removing solvent and organic in Pymol soft. Finally the key active compounds and target proteins were docked in the iGEMdock software [29, 30]. The binding degree of the receptor-ligand can be judged by the level of energy in molecular docking results. Generally, when the binding conformation of the compound and the receptor was stable, the lower the energy was, the greater the possibility of receptor-ligand interacted.

### Gene Ontology and KEGG pathway enrichment analysis

David database (https://David.Ncifcrf.Gov/summary.JSP) is an online tool for gene annotation, classification, enrichment and pathway analysis. Eventually, the key targets annotation and enriched pathways were examined by GO and KEGG pathway enrichment analysis in David database [31, 32]. p < 0.05 was used as the threshold to screen the potential signaling pathway and mechanism of hypoglycemic action of PM.

### Experimental verification

In order to verify the hypoglycemic activity of the filtered compounds, the alpha-glucosidase binding in the most common intracellular glucose metabolism during hypoglycemic biological process was selected as the index. The inhibition of alpha-glucosidase activity by the compounds was to be tested.

## Results

### Active compound and related targets

There were 187 compounds summarized forming the self-built database of PM through website database and document search. The database mainly includes stilbenes, quinones, flavonoids, phospholipids, phenylpropanoids and others. The compounds of PM are listed in the Additional file 1: Table S1. After “drug like soft” in FAFDrugs 4, 95 compounds were screened. Then 37 compounds and their predicted targets were obtained sequentially under the condition of probability value > 0.25 in STP database. In addition, a total of 1519 diabetes-related targets were acquired through the screening of disease target database. After the intersection of 37 compound-related targets and diabetes-related targets, 63 hypoglycemic targets and 29 related compounds were obtained. The 29 compounds were shown in detail in Table 2. The 63 targets were supplied in Additional file 2: Table S2.

**Table 2:** Diabetes-related key compounds in PM

No	Compound name	No	Compound name
1	Resveratrol	16	(−)-Epicatechin gallate
2	Oxyresveratrol	17	Tricin
3	Emodin	18	Apigenin
4	Aloe-emodin	19	Kaempferol
5	Rhein	20	Quercetin
6	Citreorosein	21	Luteolin
7	Teloschistin	22	Uridine
8	Emodin 8-O-β-d-glucopyranoside	23	Adenosine
9	Physcion 8-O-glucoside	24	7-Hydroxy-4-methylcoumarin-5-O-β-d-glucopyranoside
10	Emodin 1-O-β-d-glucopyranoside	25	7-Hydroxy-3,4-dimethylcoumarin-5-O-β-d-glucopyranoside
11	(−)-Catechin	26	( +)-Catechin 3-O-gallate
12	(−)-Epicatechin	27	2,6-Dihydroxybenzoic acid
13	(−)-Gallocatechin	28	3,4-Dihydroxybenzoic acid
14	(−)-Epigallocatechin	29	Gallic acid
15	( +)-Catechin gallate		

### The network of compound-targets

**Fig. 2:**
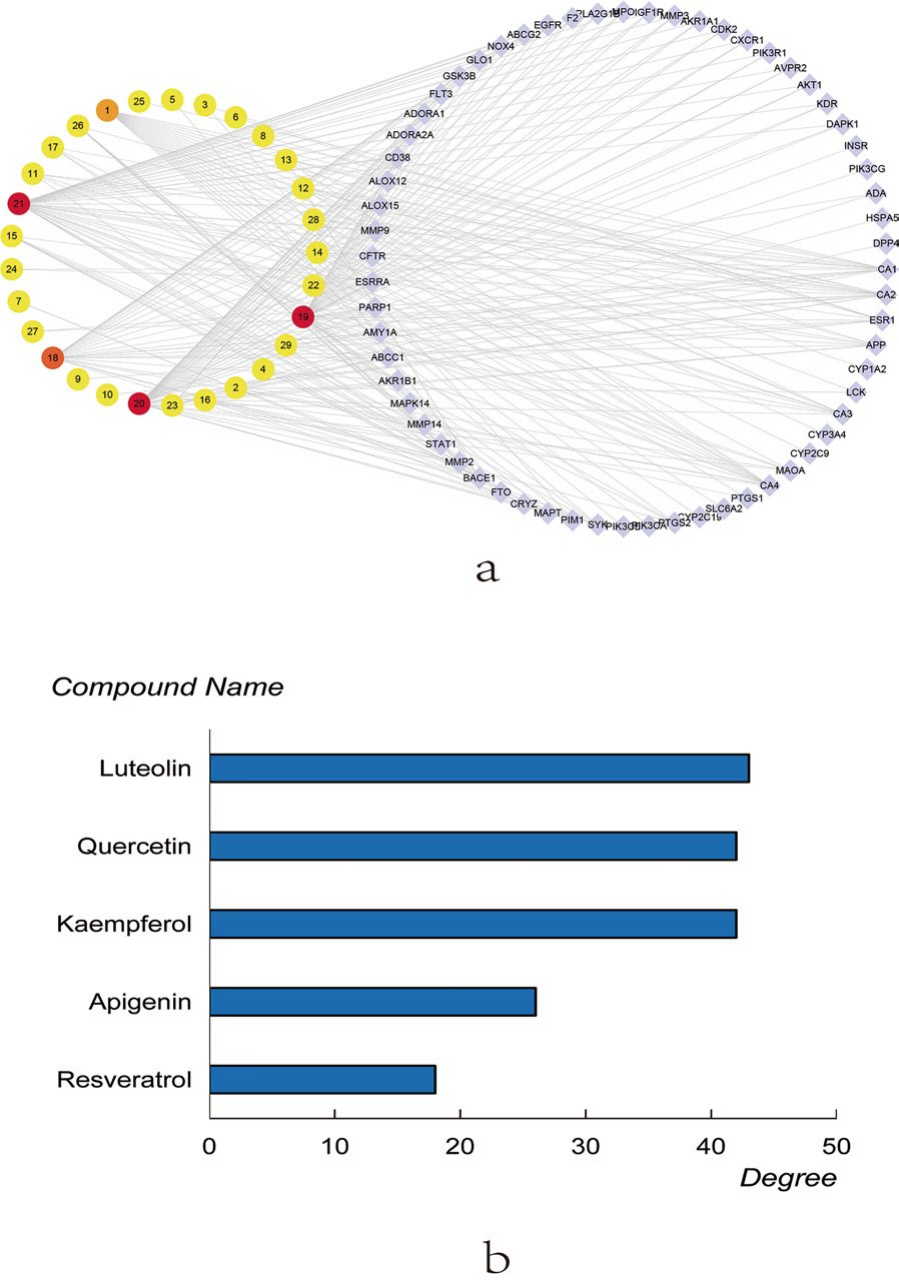
**a** The network of compound-targets interactions; **b** the top five degree compounds of PM

The topological analysis and network diagram of compound-targets were made by using Cytoscape 3.2.1. The degree values of compounds-targets were calculated. The higher the degree is, the closer the relationship between compounds and targets are, and the more important the compounds are in this network. As Fig. 2a shown, the ellipse node represents compound node and the diamond node represents the diabetes disease target. The color of node changed from yellow to red corresponds to degree from small to bigger. The numerical number represents the compound, as shown in Table 1. The target labels are indicated by the target symbols. According to the degree of the compounds, the compounds from 2 times of the average degree (17.24) to the maximum degree (43) were screened out, namely resveratrol, apigenin, kaempferol, quercetin and luteolin, respectively, as shown in Fig. 2b. What was more, ranking the target degrees, 28 targets from the average degree (3.97) to the maximum degree (15) were listed in Table 3.

**Table 3:** The information of 28 key targets ranking by degree

Target symbol	Uniprot ID	Target symbol	Uniprot ID
CA2	P00918	MAOA	P21397
CA1	P00915	ABCG2	Q9UNQ0
CA4	P22748	ALOX12	P18054
BACE1	P56817	ALOX15	P16050
CA3	P07451	CD38	P28907
ESR1	P03372	ESRRA	P11474
APP	P05067	FLT3	P36888
MMP2	P08253	GLO1	Q04760
MAPT	P10636	GSK3B	P49841
SYK	P43405	MMP9	P14780
ABCC1	P33527	NOX4	Q9NPH5
ADORA1	P30542	PARP1	P09874
ADORA2A	P29274	PIM1	P11309
AKR1B1	P15121	PTGS2	P35354

### Protein–protein interaction network

**Fig. 3:**
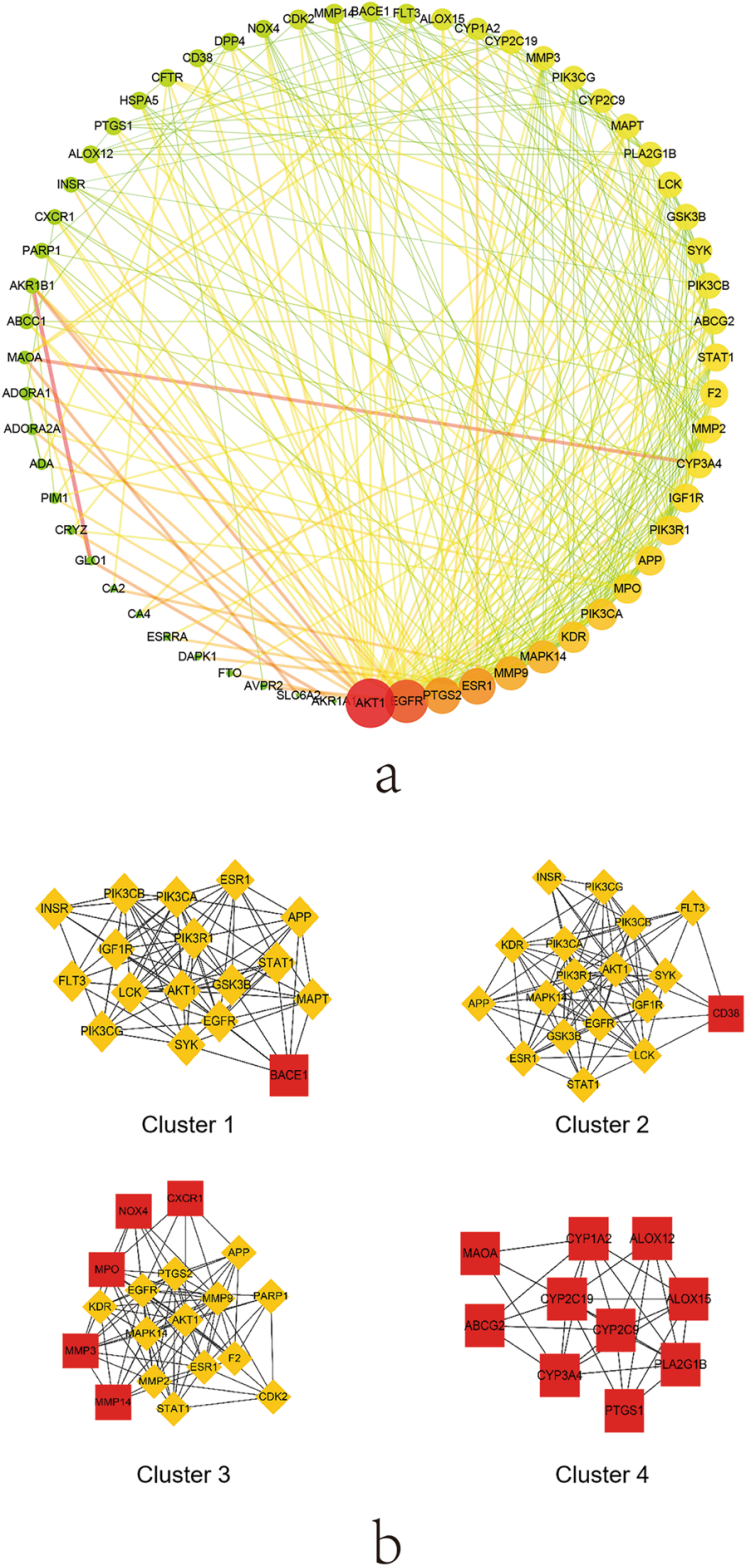
**a** The PPI network of key targets; **b** the four clustering modules of PPI network

The interaction relationship between targets was explored based on the String database. The result was shown in Fig. 3a, the target is represented by a circle node. The larger the node is, the higher the degree is and the brighter the color of the node is, the larger the betweenness centrality is. The larger and the brighter color of nodes, the more important the targets are in the hypoglycemia network of PM. The line thickness and the color depth of the nodes represent the size of the edge betweenness value. And the brighter the color of the connection line between the nodes, the thicker the line, indicates the closer the interaction relationship between the targets. At the same time, the module analysis of PPI network was carried out by using Clusterone of Cytoscape 3.2.1. There were four clustering modules with a p-value less than 0.05 among them, indicating that the target proteins in them were more closely related and may jointly perform common biological processes. As Fig. 3b shown, cluster 1 was related to the regulation of intracellular signal transduction, protein kinase B signaling and cell communication. Cluster 2 was linked with protein phosphorylation, phosphate-containing compound metabolic process and regulation of apoptotic process. Cluster 3 was connected with response to oxidative stress, regulation of reactive oxygen species metabolic process. Cluster 4 had a relation to fatty acid derivative metabolic process and oxidation–reduction process. Finally, the hub genes were screened by different algorithms and the results were specially descripted in Table 4. And it showed that AKT1, EGFR, ESR1, PTGS2, MMP9, MAPK14, and KDR were the common key targets under different algorithms.

**Table 4:** The top 10 hub genes ranked with different algorithms

Category	Rank method in CytoHubba					
	MCC	MNC	Degree	EPC	Closeness	radiality
Gene symbol top 10	AKT1	AKT1	AKT1	AKT1	AKT1	AKT1
	EGFR	EGFR	EGFR	EGFR	EGFR	EGFR
	PTGS2	ESR1	ESR1	PTGS2	PTGS2	PTGS2
	MAPK14	PTGS2	PTGS2	ESR1	ESR1	ESR1
	ESR1	MMP9	MMP9	MAPK14	MMP9	MMP9
	KDR	MAPK14	MAPK14	MMP9	MAPK14	MAPK14
	MMP9	PIK3CA	PIK3CA	KDR	KDR	KDR
	MMP2	KDR	KDR	PIK3CA	PIK3CA	APP
	MMP3	MPO	MPO	APP	APP	PIK3CA
	APP	PIK3R1	PIK3R1	IGF1R	IGF1R	IGF1R

### Molecular docking results

**Fig. 4:**
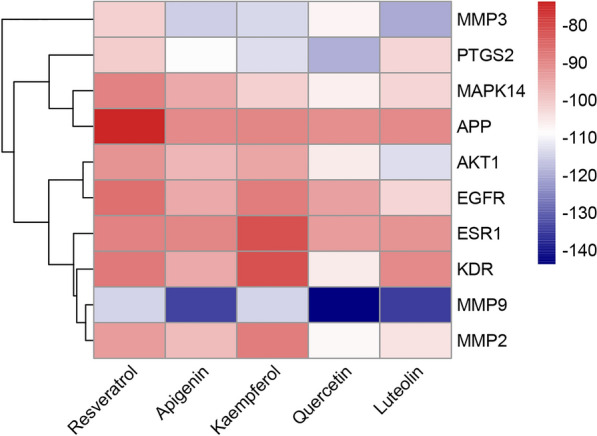
The docking energy value between ligands and receptors

The top 5 compounds by the degree ranking and the top 10 targets obtained according to the maximal clique centrality (MCC) algorithm were verified by docking systems. As Fig. 4 showed that all the docking values were all below − 73.75 Kal/mol, all of which showed low docking energy occurred between ligands and receptors. The docking energy was detailed listing in Additional file 3: Table S3. These indicated that the active components of PM could bind to the targets stably and play an effective role in diabetes. Furthermore, based on the average blinding free energy between targets and compounds, the targets ranking from low to high were MMP9, MMP3, PTGS2, AKT1, MAPK14, MMP2, EGFR, KDR, ESR1, APP. And the five compounds with MMP3, MMP9, PTGS2 were all binding with lower docking values. It means that MMP3, MMP9 and PTGS2 may be the important blinding ligands targets related to hypoglycemic effect. And the average docking values between compounds and targets shown that quercetin and luteolin are more lower among the five compounds. It indicates that quercetin and luteolin are more easier to combine with hypoglycemic activity targets.

### GO enrichment analysis of key targets

**Fig. 5:**
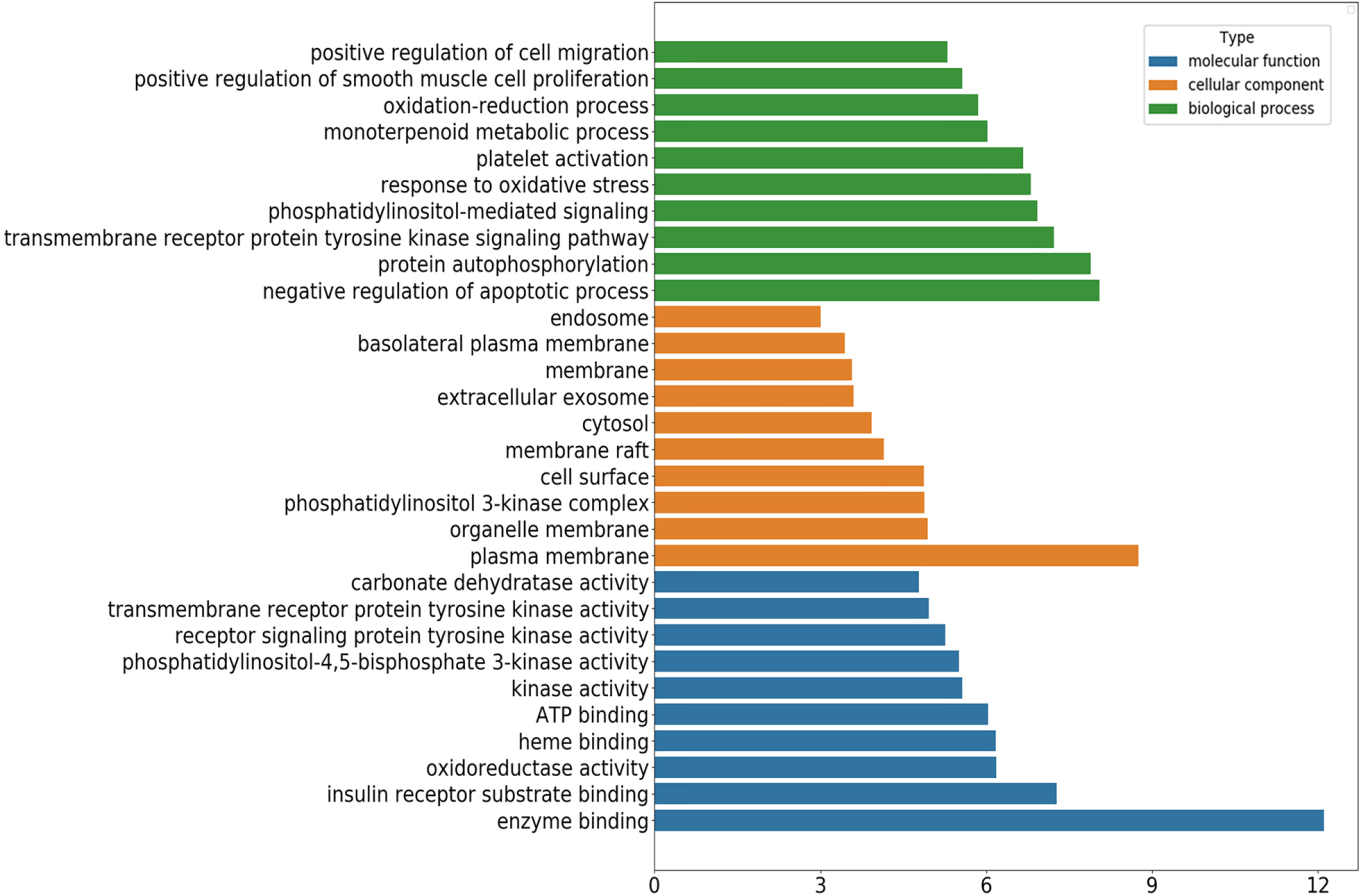
The top 10 GO terms of BP, CC and MF, respectively

DAVID website was used for GO enrichment analysis of potential targets, and a total of 208 GO terms with p < 0.05 were obtained. Among them, there were 125 entries of biological process (BP), 26 entries of cell composition (CC) and 57 entries of molecular function (MF). Figure 5 listed the top 10 most significantly enriched GO terms respectively. In the biological process category, these targets were mainly concerned with negative regulation of apoptotic process, protein autophosphorylation, transmembrane receptor protein tyrosine kinase signaling pathway, phosphatidylinositol-mediated signaling, response to oxidative stress, monoterpenoid metabolic process, oxidation–reduction process, cellular response to insulin stimulus, inflammatory response. In terms of cell composition, the targets were mainly related to membrane, cytosol, extracellular exosome and endosome. It could be seen from the biological process analysis that the targets mainly involved with enzyme binding, insulin receptor substrate binding, ATP binding, protein serine/threonine kinase activity and so on.

### KEGG enrichment analysis

**Fig. 6:**
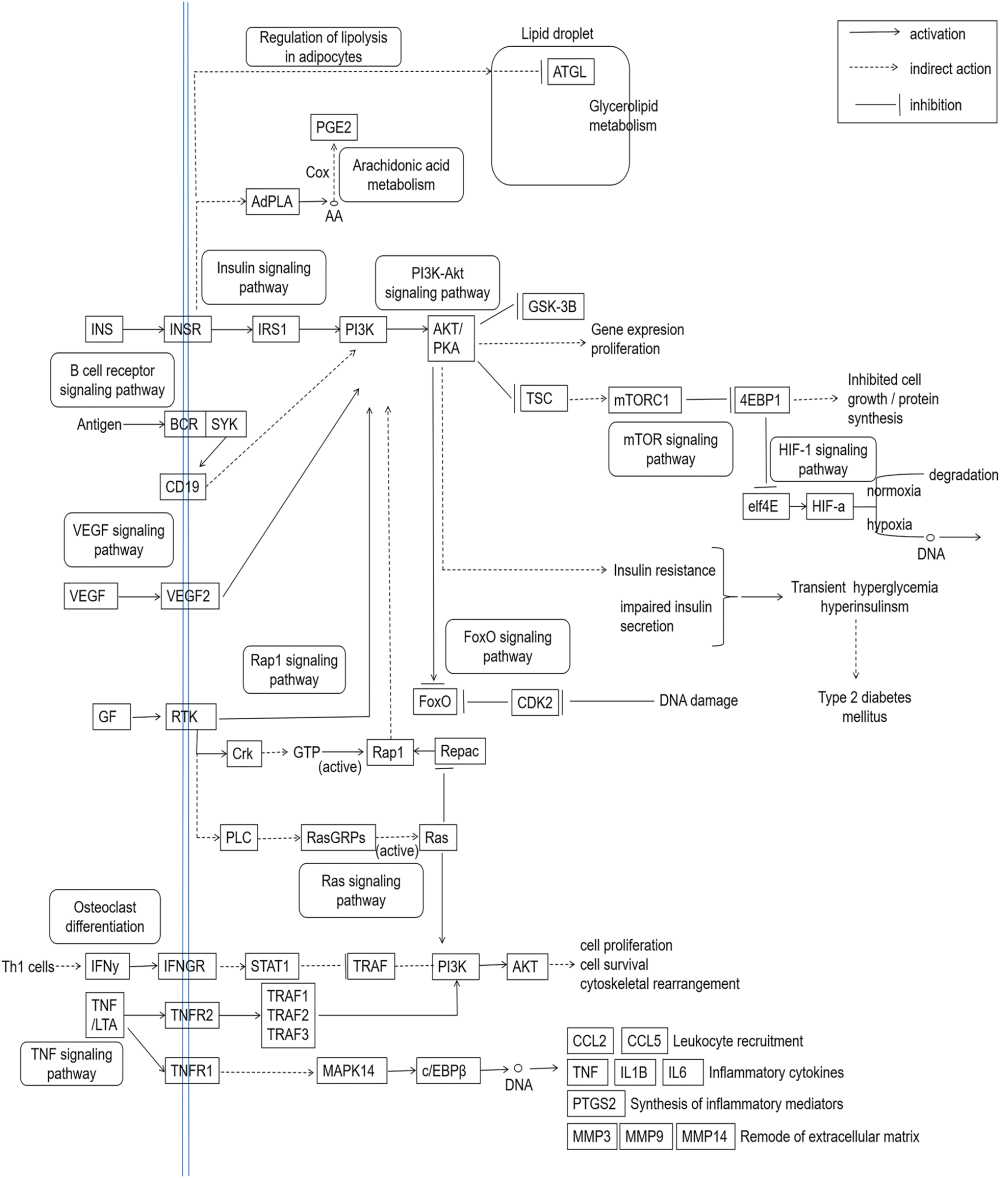
The integrated pathways of PM treating diabetes

**Fig. 7:**
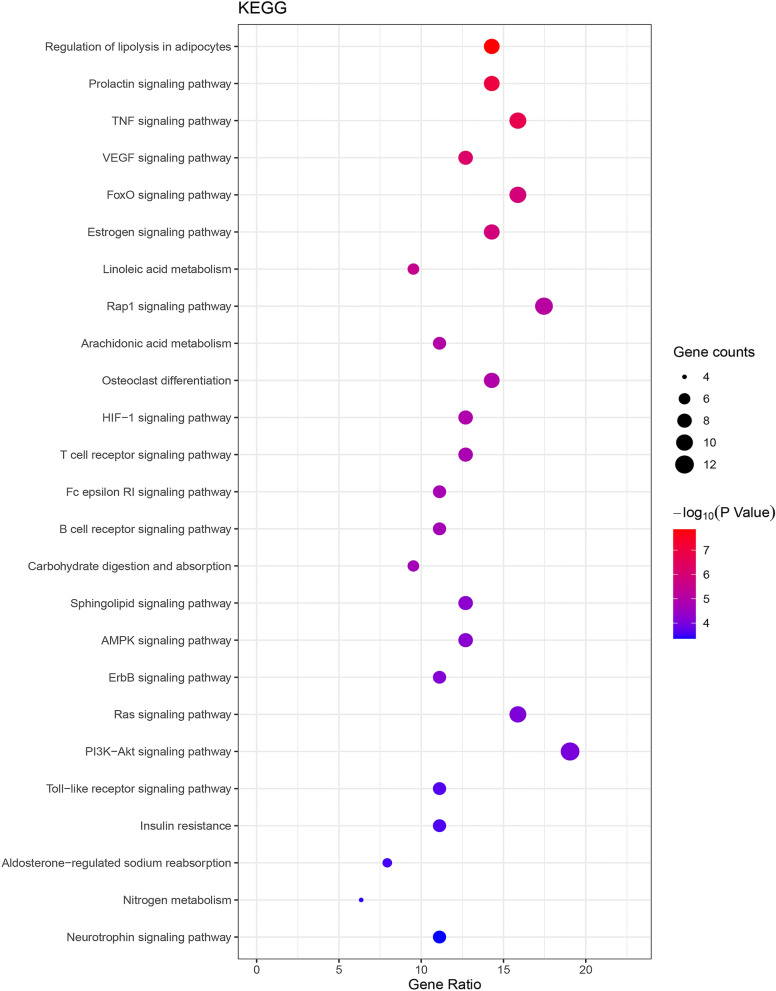
The top 25 signaling pathways of KEGG enrichment analysis

The results of KEGG pathway enrichment analysis showed that 63 hypoglycemic targets of PM were significantly enriched in 67 signaling pathways. Through the diabetes database and literature investigation, 38 pathways (Additional file 4: Table S4) were prominently related to the occurrence and development of diabetes. And the main pathways of PM treating diabetes were integrated in Fig. 6. As shown in Fig. 7, a bubble diagram was drawn by listing the top 25 signal pathways according to p value. Furthermore, the top 10 signaling pathways and their specific related genes information were shown in Table 5. Combined with p value and false discovery rate (FDR), regulation of lipolysis in adipocytes was the most significant hypoglycemic signaling pathway for PM. Particularly PI3K-Akt signaling pathway contained the most key targets.

**Table 5:** The top 10 signaling pathways with related genes

Term	Pathway	Genes
hsa04923	Regulation of lipolysis in adipocytes	PIK3CG, AKT1, PTGS2, PIK3CB, PTGS1, PIK3CA, ADORA1, INSR, PIK3R1
hsa04917	Prolactin signaling pathway	PIK3CG, AKT1, PIK3CB, MAPK14, GSK3B, ESR1, PIK3CA, STAT1, PIK3R1
hsa04668	TNF signaling pathway	PIK3CG, AKT1, PTGS2, PIK3CB, MAPK14, MMP9, PIK3CA, MMP14, MMP3, PIK3R1
hsa04370	VEGF signaling pathway	PIK3CG, AKT1, PTGS2, PIK3CB, MAPK14, PIK3CA, PIK3R1, KDR
hsa04068	FoxO signaling pathway	PIK3CG, AKT1, EGFR, IGF1R, PIK3CB, MAPK14, PIK3CA, INSR, PIK3R1, CDK2
hsa04915	Estrogen signaling pathway	PIK3CG, AKT1, EGFR, PIK3CB, MMP9, ESR1, PIK3CA, MMP2, PIK3R1
hsa00591	Linoleic acid metabolism	CYP3A4, ALOX15, CYP2C19, CYP2C9, PLA2G1B, CYP1A2
hsa04015	Rap1 signaling pathway	PIK3CG, AKT1, EGFR, IGF1R, ADORA2A, PIK3CB, MAPK14, PIK3CA, INSR, PIK3R1, KDR
hsa00590	Arachidonic acid metabolism	ALOX15, CYP2C19, PTGS2, CYP2C9, PTGS1, PLA2G1B, ALOX12
hsa04380	Osteoclast differentiation	PIK3CG, AKT1, PIK3CB, MAPK14, LCK, PIK3CA, STAT1, PIK3R1, SYK

### Experiment results

**Fig. 8:**
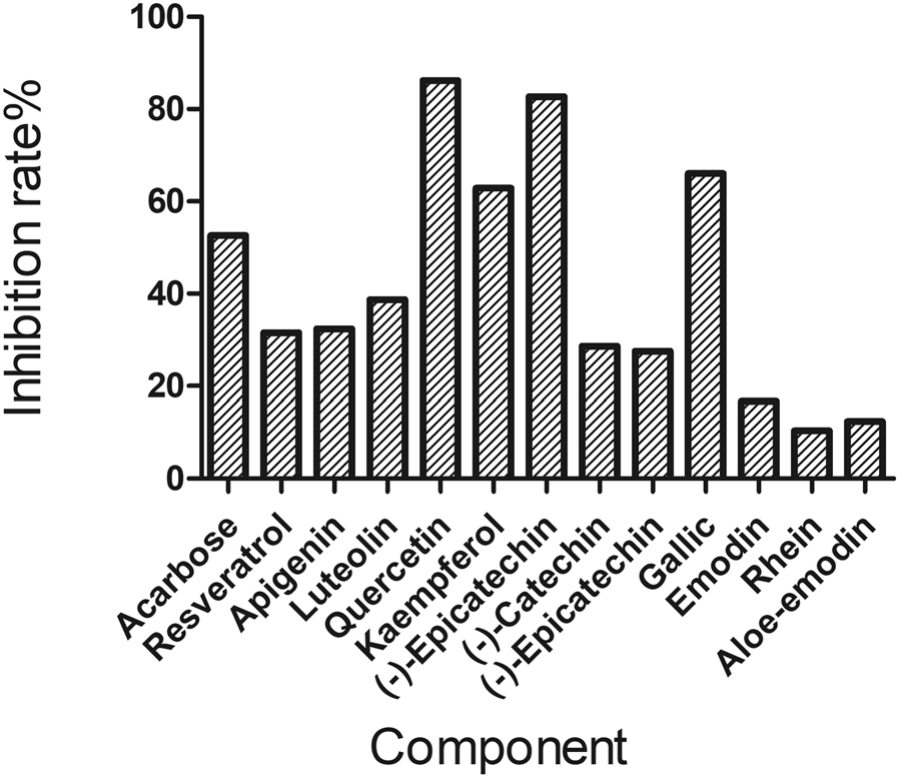
The alpha-glucosidase inhibition rate of part of potential active ingredients

The inhibitory effect of part of screened compounds on alpha-glucosidase activity was detected to evaluate the hypoglycemic activity. Acarbose (20 µg/ml) was selected as the positive control, and the concentration of the compounds to be tested were 10 µM to evaluate the inhibition rate on alpha-glucosidase activity. The result was shown in Fig. 8. The alpha-glucosidase inhibition rate of resveratrol, apigenin, luteolin, quercetin, kaempferol, (−)-epicatechin gallate, (−)-catechin, (−)-epicatechin, gallic acid, emodin, rhein and aloe-emodin were 52.6%, 31.5%, 32.4%, 38.7%, 86.2%, 62.9%, 82.7%, 28.6%, 27.5%, 66%, 16.7%, 10.4% and 12.4%. They all showed great inhibitory activity. Especially for the top 5 compounds, the inhibition rate of resveratrol, apigenin, luteolin, quercetin and kaempferol were all no less than 30%. The experimental results showed that the screened compounds have good hypoglycemic activity, which could be potential active ingredients.

## Discussion

Based on the evaluation of druggability and network pharmacology screening, 29 components of PM had a potential hypoglycemic effect. Among them, there were 2 stilbene glycosides, 8 quinones, 11 flavonoids, 2 nucleosides, 2 coumarins and 4 others. According to the analysis of sequential compound-target network, the top five compounds contained one stilbene glycoside and four flavonoids, namely resveratrol, apigenin, kaempferol, quercetin and luteolin. Resveratrol (3,5,4′-trihydroxy-trans-stilbene), which was reported inhibiting CCR6-mediated migration of inflammatory cells, may have a promising treatment with T1DM [33]. Furthermore lots of experiments and clinical trials have shown that resveratrol had beneficial effects on T2DM by improving glucose homeostasis, decreasing insulin resistance, protecting pancreatic beta-cells, improving insulin secretion and ameliorating metabolic disorders [34–37]. Studies also suggested that resveratrol could increase the activity of AMPK and SIRT1, decreasing adipogenic genes and provide some promising mechanisms [38, 39]. Wang commented on the effect of apigenin on streptozotocin (STZ) induced pancreatic β cell damage diabetes model. The results showed that apigenin was not only increasing serum insulin and pyruvate kinase, but also regulating to antioxidant defenses of pancreatic cells [40, 41]. Besides studies suggested that apigenin was sensitive to diabetes by mediating MAPK pathway Regulating ROS/caspase-3 and NO pathway [42, 43]. Kaempferol, another flavonoid screened, has been proved to have good activity on T2DM and its complications. It was reported that Kaempferol could enhance insulin sensitivity and inhibit hepatic gluconeogenesis to treat with T2DM [44, 45]. In vivo studies have shown that kaempferol down-regulated IKK and suppressed NF-kappa B pathway activation to reduce hepatic inflammatory lesions, which was helpful to insulin signaling defect in diabetes [46]. And Bhatia found kaempferol ameliorated myocardial injury in diabetic rats through suppressing mitogen activated protein kinase (MAPK) pathway and activating AGE-RAGE axis [47]. What is more, in vitro and in vivo evidence have reviewed for quercetin treatment with diabetes and its complications [48, 49]. Research results suggested that quercetin was probably associated with SIRT1/NLRP3 pathway and Akt signaling pathway [50, 51]. Hence, quercetin showed potential for the treatment of glucose and lipid metabolism disorder in diabetes mellitus. Dhanya found that quercetin may correct the insulin resistance through the regulated system for GLUT4 translocation, which was connected with adenosine monophosphate kinase (AMPK) pathway and its downstream target p38 MAPK [52]. Another study showed that ERK1/2 played a major role in quercetin for preventing beta-cell dysfunction [53]. Study also showed luteolin could improve the cardiac function in diabetic rats due to reducing mitochondrial oxidative stress and mitochondrial swelling [54, 55]. Another research suggested that luteolin could ameliorate the cognitive dysfunctions in STZ-induced diabetic rat model by downexpression of glycation end products (AGEs), inhibition of IL-1 and TNF- and upregulating the expressions of GAP-43 and SYN [56]. In addition, Xu caught sight of mast cells playing a key role in diet-induced obesity and diabetes for reducing mast cell and macrophage infiltrations and inflammatory cytokine levels and inhibiting mast cell-derived IL-6 expression [57].

Resveratrol have been wildly used for its various of beneficial properties. The rapid elimination and highly effective absorption of resveratrol have been a big issue of bioavailability [58]. Pharmacokinetics studies of resveratrol have been well established in animals and in humans. Measurement of the urinary excretion data indicated a 70% absorption rate after oral consumption of resveratrol. The half-life times of plasma were 9.2 h after oral administration and 11.4 h after intravenous injection in humans [59]. When lower dose (5–50 mg) was given, resveratrol glucuronides are the prominent metabolites in plasma. When higher dose (> 250 mg) was given, monosulfates are the main metabolites. It shows a concentration-dependent biotransformation [60]. In addition, blood kinetics showed a high elimination half-time (91.8 h), a distribution volume of 259 ml, and a plasmatic clearance of 1.95 ml/h for apigenin in the rat. All the pharmacokinetic parameters showed a slow metabolism of apigenin, with a slow absorption and a slow elimination phase. Thus, a possible accumulation of apigenin may occur in the body [61]. kaempferol followed a one-compartment model, with a rapid clearance (4.40–6.44 l/h/kg) and an extremely short half-life of 2.93–3.79 min after intravenous administration. After oral gavage it was not possible to obtain full plasma concentration–time profiles of kaempferol. Plasma exposure of kaempferol is limited by poor oral bioavailability and extensive metabolism [62]. After absorption, kaempferol is extensively metabolized in the liver to form glucurono- and sulfo-conjugated forms [63]. Some studies have found that kaempferol was metabolized to quercetin and that this effect was probably mediated by the enzymes CYP1A. some of the effects induced by kaempferol in vivo might be mediated in part by quercetin, which is known to have a wide range of biological activities [64]. Quercetin is a natural flavonoid, quercetin (Qr), isoquercitrin (IQ), and quercetin-3-O-β-d-glucuronide (QG) were also mutual transform in vivo. The t1/2 of quercetin is about 437 min [65]. And studies have shown that QG is a major active component in plasma and tissue for oral administration of Qr or QG [66]. Luteolin have the same poor oral bioavailability at 26 ± 6% [67]. There are multiple peaks at a Tmax of 0.5 h and at 4 h after oral administration of luteolin (100 mg/kg, po), which suggested that enterohepatic circulation is a general phenomenon [68]. In summary, the five compounds showed significant pharmacokinetic differences. Furthermore they showed slower metabolism, low bioavailability and extensive metabolic transformation, which reflects the synergistic effect of hypoglycemic effects from other side.

The ingredient-target network showed the characteristics of multi-component and multi-target of hypoglycemic in PM. The PPI network illustrated that there was a correlation among the target proteins of PM. The further analysis of PPI results indicated that the hub genes AKT1, EGFR, ESR1, PTGS2, MMP9, MAPK14, and KDR were the common targets of the top 10 in different algorithms. These targets may be the main targets of PM to diabetes mellitus. AKT was responsible for the regulation of glucose uptake by mediating insulin-induced translocation of the SLC2A4/GLUT4 glucose transporter to the cell surface. AKT also regulated the storage of glucose in the form of glycogen and mediated insulin-stimulated protein synthesis (PubMed: 12150915). EGFR was associated with activating several signaling cascades to convert extracellular cues into appropriate cellular responses (PubMed: 2790960). Research suggested that the association between T2DM and ESR1 variants may be because of other unidentified ESR1 polymorphisms that regulated glucose homeostasis [69]. PTGS2, which negatively modulated glucose-stimulated insulin secretion, and functioned as a mediator of the inflammatory response, associated with decreased insulin sensitivity, may play a role in mediating susceptibility to T2DM [70]. Study also suggested that MMP9 might connect with autophagy mediated contractile dysfunction in diabetes [71, 72]. MAPK14 and other proteins, which played crucial roles in the regulation of glucose metabolism, were all significantly down-regulated in the skeletal muscle of diabetic rats and insulin-resistant L6 cells [73]. Khazaei’s results demonstrated that reduced serum nitric oxide and vascular endothelial growth factor receptor 2 levels may be responsible for the decreased myocardial capillary density in diabetic rats [74]. In summary, the key targets were of great significance in diabetes and constructed a target-interaction network for the antidiabetic effect of PM. What’s more, it provided a scientific basis for elaborating the mechanism of PM to hypoglycemic effect through multiple targets and multiple pathways.

Diabetes is a complex endocrine system disease involving many metabolic pathways. After KEGG enrichment analysis of key targets, the following top 10 disease-related metabolic pathways were obtained. Regulation of lipolysis in adipocytes: lipolysis played a crucial role in obesity-related insulin resistance, and modulating lipolysis in adipocytes may lead to a new treatment to diabetes [75, 76]. Prolactin signaling pathway: The functions of prolactin receptor has been extended to include islet differentiation, adipocyte control and immune modulation. Also the prolactin signaling pathway disruption has been involved in diabetes [77]. TNF signaling pathway: tumor necrosis factor-alpha (TNF-α), one of the most crucial pro-inflammatory mediator, induced insulin resistance in adipocytes by impairing the insulin signaling through serine phosphorylation so that leading to the development of T2DM [78]. VEGF signaling pathway: Previous reports indicated that ROS-induced VEGFR2 signaling might be a promising treatment of endothelial dysfunction and serious vascular diseases in diabetes [79]. FoxO signaling pathway: FoxO proteins, which could regulate insulin signaling, gluconeogenesis, insulin resistance, immune cell migration, and cell senescence, have become new clinical entities to treat metabolic disorders and diabetes mellitus [80]. Estrogen signaling pathway: studies have demonstrated that augmenting liver estrogen signaling through ER-α may prevent hepatic insulin resistance and lessen the impact of obesity on diabetes and cardiovascular risk in male rats [81]. Linoleic acid metabolism: reports have shown that the improvements of diabetes could increase the desaturation levels of linoleic acid and its desaturation products in various body compartments [82]. Rap1 signaling pathway: some studies indicated that activated Rap1A promoted glucose-stimulated insulin secretion, islet cell hypertrophy, and islet cell proliferation, and it can be a unique target for the treatment of beta-cell dysfunction in diabetes [83]. Arachidonic acid metabolism: the altered arachidonic acid metabolism and the increased ratio of thromboxane A2 to prostacyclin, which favored an enhanced thrombotic state, may play a role in the accelerated vascular disease of diabetes mellitus [84]. Osteoclast differentiation: it was reported that T2DM could inhibit osteoclastogenesis and osteoclast activity, and delay the degradation of matrix during the alveolar bone regeneration in T2DM rats [85].

In summary, the study clearly illuminated the action mode of multi-component and multi-target of PM in the therapeutical effect on diabetes mellitus. According to KEGG analysis results, PI3K-Akt signaling pathway was the most enriched signaling pathway. And the integrated signaling pathway map showed that the PI3K-Akt axis was at the center of all related pathways. This indicated that the PI3K-Akt signaling pathway may be associated with Hypoglycemic effect as the most critical pathway. In addition, a total of 29 components in PM were screened out as the key active components of hypoglycemic, and the most significant 5 key components were verified by molecular docking and enzyme activity inhibition tests. However, there were still some limitations to this study. the result was only based on data mining and network pharmacology analysis. Many unidentified components of PM were still not included in it. In vivo hypoglycemic test verification need further evaluation.

## Conclusion

Based on the mode of action of multi-component and multi-target for traditional Chinese medicine and in line with the principles of network pharmacology, this study systematically explored the bioactive components and mechanism of the hypoglycemic effect of PM. The network interaction diagram of 29 key hypoglycemic components of PM was constructed, corresponding to 63 key hypoglycemic action targets. Furthermore, resveratrol, apigenin, kaempferol, quercetin and luteolin, the five uppermost hypoglycemic components were selected. In addition, the molecular docking method and enzyme activity inhibition tests verified the good activity of the active compounds against diabetic targets. Finally it was found that 38 functional pathways were involved in PM glucose-lowering through data mining and network analysis. As a whole, this study provides reference and scientific basis for further study on the hypoglycemic effect of PM or its active ingredients and the secondary development of PM.

## Supplementary information

**Additional file 1.** The compounds database of PM.

**Additional file 2.** The hypoglycemic targets related to PM.

**Additional file 3.** The specific values of docking energy between ligands and receptors.

**Additional file 4.** The detailed information about 38 related pathways.

## Data Availability

The datasets used and/or analyzed during the current study are available from the corresponding author upon reasonable request.

## Abbreviations

TCM: Traditional Chinese medicine
PM: Polygonum multiflorum
PPI: Protein–protein interactions
GO: Genome ontology
KEGG: Kyoto encyclopedia of genes and genomes
T1DM: Type 1 diabetes mellitus
T2DM: Type 2 diabetes mellitus
IDF: International diabetes federation
STP: Swiss target prediction
SEA: Similarity ensemble approach
TTD: Therapeutic target database
PDB: Protein data bank
